# Case report: Diagnostic challenges in early onset Alzheimer’s disease

**DOI:** 10.1192/j.eurpsy.2023.2124

**Published:** 2023-07-19

**Authors:** E. Chabukovska, S. Todevska, O. Pashanko, M. Petrushevska, G. Novotni, A. Novotni, V. Sukloska

**Affiliations:** 1 Adult psychiatry, Psychiatric hospital Skopje, Skopje; 2 Adult psychiatry, PHI Clinical Hospital “Dr. Trifun Panovski”, Bitola; 3 Adult psychiatry, PHI Health Center; 4 Neurology, University Clinic of Neurology; 5 Adult psychiatry, University Clinic of Psychatry, Skopje; 6 Adult psychiatry, PHI Psychiatric Hospital “Demir Hisar”, Demir Hisar, North Macedonia

## Abstract

**Introduction:**

According to World Alzheimer Report 2021, more that 55 million of people in the world suffer from dementia. And although age is the strongest known risk factor for dementia, dementia does not exclusively affect older people. Early onset dementia is defined as the onset of symptoms before the age of 65 years. Considering that people with early onset dementia are in the most productive period of their life and often exposed to stress, when the symptoms of depression or psychosis can appear linked to other psychiatric diagnose it is hard to think of dementia when it is in early stage.

**Objectives:**

We present a case of a woman at age of 55, mother of one child, widow, with secondary school degree, employed as textile worker.

She was already on psychiatric treatment for five years diagnosed at first as Mixed anxiety and depressive disorder and after that as Major depressive disorder, single episode, severe with psychotic features. Her past treatment include Sertraline up to 100mg per day or Escitalopram up to 10mg per day and Olanzapine up to 10mg per day. But her condition was worsening progressive with cognitive decline and during serial stressful events in the family (the death of her husband and severe corona virus infection of her son).

At present time she was hospitalized with psychotic symptoms, confusion, paranoid ideas and hallucinations, dysfunctional in everyday activities.

**Methods:**

The neuropsychological testing showed global reduction in cognitive-behavior status. The results of extended laboratory tests were in normal range. Brain MRI showed global cortical reduction with more specified atrophy in fronto-temporal lobes bilateral. SPECT analysis showed significant hypoperfusion in both hemispheres in frontal, parietal and temporal lobes. Cerebrospinal fluid examination showed decresed level of beta-amyloid-42 (281,6 pq/ml).

**Results:**

The results confirmed the diagnose of dementia with early onset, but because of advanced stadium and insufficient family history it was not possible to make clinical diagonose of the type. Diagnose in the end of hospitalization was: Early onset dementia, M. Alzheimer frontal variant.

**Conclusions:**

With the presented case we suggest that the clinicians need to be very careful in the cases of psychosis treated independently and explore the possibility that psychosis can be a symptom of Alzheimer desease. Our case suggest that we should consider the possibility of early onset AD in middle-aged patients whose first symptoms are depressive with psychotic features. In this respesct, psychiatris need to consider proper completion of AD diagnostic protocol including biomarkers analysis.

**Disclosure of Interest:**

None Declared

